# The severity of adverse drug reactions and their influencing factors based on the ADR monitoring center of Henan Province

**DOI:** 10.1038/s41598-021-99908-3

**Published:** 2021-10-14

**Authors:** Ziqi Yan, Zhanchun Feng, Zhiming Jiao, Chaoyi Chen, Ganyi Wang, Da Feng

**Affiliations:** 1 School of Medicine and Health Management, Tongji Medical College, Huazhong University of Science and Technology, Wuhan, 430030 Hubei China; 2 Medical Products Administration and Center for ADR Monitoring of Henan, Zhengzhou, 450008 Henan China; 3 School of Pharmacy, Tongji Medical College, Huazhong University of Science and Technology, Wuhan, 430030 Hubei China

**Keywords:** Health care, Risk factors

## Abstract

Adverse drug reactions (ADRs) may be a serious public health problem and have received widespread attention in recent years. This study has analyzed the factors leading to the occurrence of serious ADRs (SADRs), determined the factors affecting the prognosis of patients with severe adverse reactions at different levels of medical institutions, and finally made corresponding recommendations for the monitoring, prevention, and treatment of SADRs. We used descriptive analysis and chi-square test to analyze the year, age, gender, proportion of SADRs, and the results of the ADRs in the report. Use the logistic regression to analyze the factors affecting the prognosis of SADRs in different levels of medical institutions. A total of 387 642 people’s 394 037 ADRs were collected from the Henan Provincial Adverse Drug Reaction Monitoring Center from 2016 to 2020. Among them 35 742 cases of serious ADRs (9.1%), 96.1% were eventually relieved or cured, but 39 cases of SADRs caused death. The main causes of death included hemorrhages, organ failure, and allergies. Age, number of medication and illnesses, level of medical institution, history of adverse reactions, and type and method of medication were all factors that affected the severity of ADR. The prognosis of SADRs is worse than normal ADRs. The ADRs in autumn and winter and new adverse reactions are unique risk factors found in this study. The elderly and patients with multiple diseases or taking multiple drugs should pay attention to their adverse reactions. They should be closely observed within a week after taking the medicine. The supervision of patients with a history of allergies and new adverse reactions should be strengthened by primary medical institutions, and in nonprimary medical institutions should paid attention with past medical histories, and use imported drugs and biological agents with caution to ensure the safety and health of patients.

## Introduction

Given the increased development and utilization of drugs, adverse drug reactions (ADRs) have gradually become a public concern^1,2^. ADRs refer to unrelated or unexpected adverse reactions of qualified drugs under normal usage and dosage^3^. Serious ADR (SADR), a heterogeneous reaction completely unrelated to normal pharmacological effects, cannot be detected by conventional toxicological screening, has a low incidence, and is delayed, not dependent on dose, and unpredictable^4^. Once SADRs occur, multiple organs throughout the body are involved, seriously threatening the life and safety of patients.

SADRs seriously threaten the lives and health of patients and cause a lot of waste of medical resources. From 1966 to 1996, in the United States, an average of 6.7 in every 100 hospitalized patients have SADRs, and the mortality rate reaches 0.32%^5,6^. The average length of stay of each hospitalized patient due to ADRs is extended by two days, and the average cost increases by $2500^7,8^. In recent years, the number of ADR reports in China has increased rapidly. The China Adverse Drug Reaction Monitoring System has received 1.676 million ADR reports in 2020 (1251 cases per million population), and SADRs account for 10% of these reports^9^. SADRs increase the cost of medical treatment for patients, may delay the treatment time of patients, and seriously affect the quality of life of patients. SADRs also cause patients to lose trust in doctors, causing both parties to fall into medical disputes and aggravating the tense doctor–patient relationship. SADR has become one of the main factors that increase the uncertainty of clinical drug research and development and may terminate research and development due to damage to the health of patients^10^. Thus, SADRs affect the health of patients and adversely affect the operation of medical institutions.

China has introduced the *Adverse Drug Reaction Reporting and Monitoring Provision* in 2010^11^. By 2015, more than 280 000 are registered users of the China Adverse Drug Reaction Monitoring System (an online spontaneous reporting system). Users include pharmaceutical manufacturers, drugstores, and medical institutions, and nearly 16.87 million ADR/event reports are collected from 1999 to 2020^9^. The ADR reporting and monitoring have developed rapidly, and report numbers and reporting rates are increasing^12^, providing data support for this research.

Therefore, this study has analyzed the factors leading to the occurrence of SADRs, determined the factors affecting the prognosis of patients with severe adverse reactions at different levels of medical institutions, and finally made corresponding recommendations for the monitoring, prevention, and treatment of SADRs.

## Methods

### Data source and preprocessing

The data of the adverse drug reaction reports collected by Adverse Drug Reaction Monitoring Center of Henan Province from January 2016 to December 2020 were classified and analysed, and spontaneously reported by medical institutions, enterprises, and the public in Henan.

Data were cleaned and preprocessed to ensure that they were clean and complete. A total of 571 326 initial data were obtained. Everyone will record a code once an ADR occurs, but there are cases where the same code includes multiple entries of different drugs. We use excel to ensure that each code retains one to eliminate duplicate data, and 394 037 records of ADRs were retained.

### Data analysis

The year, age, gender, proportion of serious adverse reactions, and the results of the adverse reactions in the report were subjected to descriptive analysis and chi-square test. The logistic regression was used to analyze the factors affecting the prognosis of SADRs in different levels of medical institutions. All data analyses were performed using the SPSS 24.0 software (IBM Corp. Armonk, NY). A p-value less than 0.05 was considered statistically significant.

### Variable assignment

The variables analyzed all came from the *Adverse Drug Reaction Event Report Form*. This research encoded and assigned variables in accordance with the *Adverse Drug Reaction Reporting and Monitoring Management Measures*^13^*.*

This study based on the regulations of the National Adverse Drug Reaction Monitoring Center, Among the reported ADRs, Death; teratogenic, carcinogenic, or birth defect; permanent sequelae; permanent damage to organ function; leading to hospitalization or prolonged hospital stay were regarded as “Serious ADRs” (Abbreviated as SADR), other cases were regarded as “Normal ADRs”.

About the distinction of medical institutions, This study refers to *The measures for the administration of the hospital grade* issued by the Ministry of Health institutions that mainly provide basic public health services and basic medical services as primary hospitals, and other comprehensive medical institutions as non-primary hospitals in accordance with relevant national policies. All primary institutions are first-level hospitals, secondary hospital is a regional hospital that provides comprehensive medical and health services to multiple communities and undertakes certain teaching and scientific research tasks, tertiary hospitals are usually provincial and municipal hospitals.

### Outcome definition

The study outcomes were ADR results and the impact on the original disease. In the binary logistic regression, we defined patients who had recovered from ADRs and had no significant impact on the pre-existing disease as a good prognosis, and defined patients who did not improve or had a worsening of the original disease as a poor prognosis.

### Ethics

The study protocol was reviewed. Ethical approval was obtained from the Ethics Committee of Tongji Medical College, Huazhong University of Science and Technology (2020S204).

This study obtained written informed consent statements from all human participants, and obtained the written informed consent statements of Ganyi Wang, the legally authorized representative of the minor participants.

The study protocol is performed in accordance with the relevant guidelines.

## Results

### Demographic characteristics of ADRs

**Figure 1:**
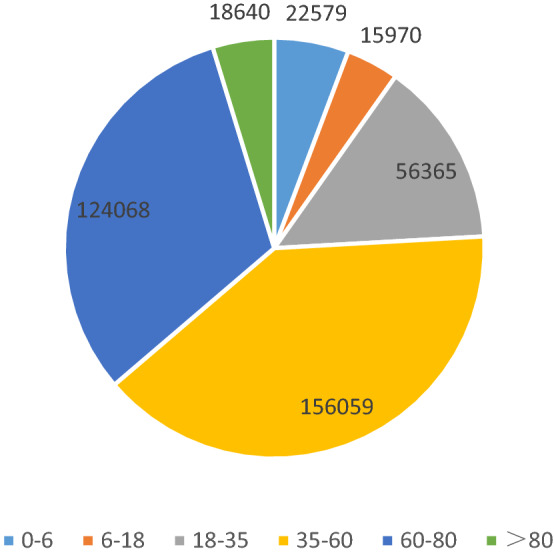
Number of ADR age from 2016 to 2020.

**Figure 2:**
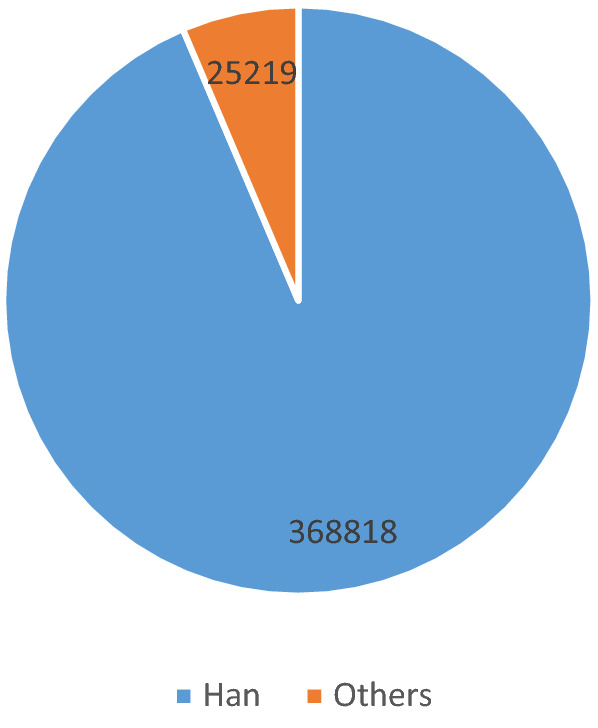
Number of ADR nations from 2016 to 2020.

Among the 394 037 reports of ADRs, Figs. 1 and 2 shows 52.3% of the patients are women (206 042), and the rest are men (187 473). The difference between genders is not significant. About 93.6% of patients are of Han nationality, and 36.3% of patients are older than 60 years old. 1.46% people (5673) had 2 or more ADRs.

### Occurrence of ADRs

According to the occurrence of ADRs in the medication process, 60.5% (238 545) of ADRs occur on the day of medication. About 94.7% of ADRs occur within one week of medication, and only 0.9% of adverse reactions occur after one month.

### Reports about ADRs

**Figure 3:**
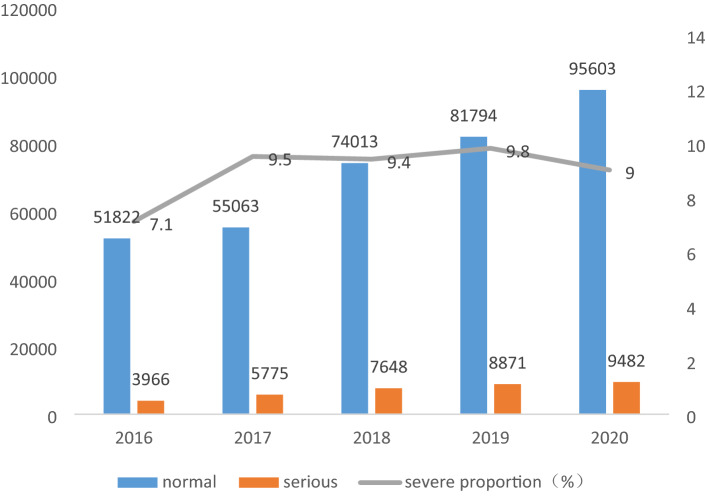
Number of ADR cases from 2016 to 2020.

Figure 3 shows 394 037 ADRs in 2016–2020. Both the number of reported ADRs and the proportion of severe ADRs have increased since 2016.

**Figure 4:**
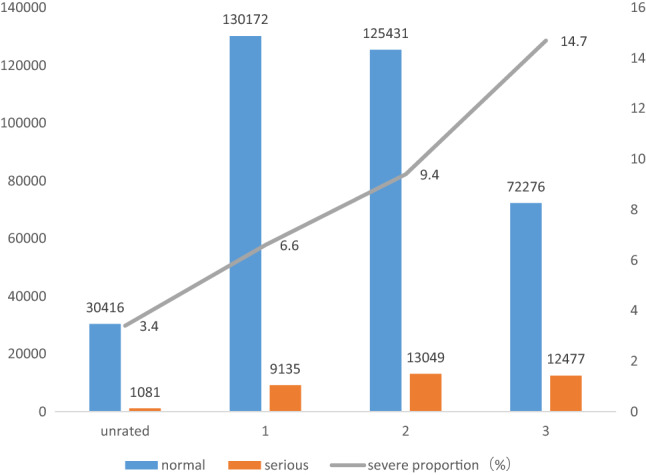
The proportion of SADRs in different levels of medical institutions.

Figure 4 shows the number of ADRs and the proportion of SADRs in different levels of medical institutions. A high hospital level results in high proportion of SADRs.

### Severity of ADR in patients with different characteristics

Table 1 summarizes the SADRs in patients with different characteristics. Severity is not related to gender but related to other factors. Underaged and elderly patients, third-level hospitals, patients with new and recurring ADRs, ADRs in autumn and winter, and patients who take drug injections or used imported and biologic durgs have a high proportion of SADRs.

**Table 1:** The severity of adverse drug reactions was different in patients with different characteristic.

Variables	Total N (%)	Nonserious N (%)	Serious N (%)	*P* value	Effect size (φ)
Total	394,037 (100)	358,295 (90.90)	35,742 (9.10)		
**Age (years)**					
0–6	22,579 (5.73)	20,888 (92.51)	1691 (7.49)	< 0.001	0.560
6–18	15,970 (4.05)	14,718 (92.16)	1252 (7.84)		
18–35	56,365 (14.30)	52,659 (93.42)	3706 (6.58)		
35–60	156,059 (39.61)	142,930 (91.59)	13,129 (8.41)		
60–80	124,068 (31.48)	110,619 (89.16)	13,449 (10.84)		
> 80	18,640 (4.73)	16,468 (88.35)	2172 (11.65)		
Missing	356 (0.10)				
**Gender**					
Male	187,473 (47.58)	170,404 (90.90)	17,069 (9.10)	0.496	-0.001
Female	206,042 (52.29)	187,411 (90.96)	18,631 (9.04)		
Missing	522 (0.13)				
**Hospital level**					
Unrated	31,497 (7.99)	30,416 (96.57)	1081 (3.43)	< 0.001	0.119
1	139,307 (35.35)	130,172 (93.44)	9135 (6.56)		
2	138,480 (35.14)	125,431 (90.58)	13,049 (9.42)		
3	84,753 (21.50)	72,276 (85.28)	12,477 (14.72)		
**Medication method**					
Oral	165,060 (41.89)	159,655 (96.72)	5405 (3.27)	< 0.001	0.180
Injection	218,327 (55.41)	188,392 (86.29)	29,935 (13.71)		
Others	10,650 (2.70)	10,248 (96.23)	402 (3.77)		
**Type of drug**					
Chemical compound	302,078 (76.66)	273,904 (90.67)	28,174 (9.33)	< 0.001	0.410
Chinese patent medicine	72,218 (18.33)	66,777 (92.47)	5441 (7.53)		
Imported and biologics	12,163 (3.09)	10,415 (85.63)	1748 (14.37)		
**Past history**					
No	257,061 (65.24)	233,255 (90.74)	23,806 (9.26)	< 0.001	0.570
Yes	5922 (1.50)	4648 (78.49)	1274 (21.51)		
Unknown	131,054 (33.26)	120,392 (91.86)	10,662 (8.14)		
**ADRs occurrence**					
First	387,642 (98.38)	352,672 (90.98)	34,970 (9.02)	< 0.001	0.013
Again	6395 (1.62)	5623 (87.93)	772 (12.07)		
**New ADRs**					
Yes	101,727 (25.82)	91,208 (89.66)	10,519 (10.34)	< 0.001	0.026
No	292,310 (74.18)	267,087 (91.37)	25,223 (8.63)		

### Effect of ADRs

Table 2 shows the results of ADR about different severities and the effects on the original disease. For normal ADRs, 98% of the cases eventually got better or cured and there was no death.However, for SADRs, only 76.2% of the cases got better. The proportions of unimproved, worsening, and sequelae are all higher than the group of normal ADRs, and all 39 deaths were also from SADRs group.

**Table 2:** The severity of ADRs was different in patients with different characteristics.

Variables	Total N (%)	Nonserious N (%)	Serious N (%)	*P* value	Effect size (φ)
Total	394,037 (100)	358,295 (90.58)	35,742 (9.42)		
**Results**					
Better	378,497 (96.06)	351,276 (98.00)	27,221 (76.20)	< 0.001	0.332
Not better	13,066 (3.31)	5486 (1.50)	7580 (21.20)		
Worse	1720 (0.44)	1302 (0.40)	418 (1.20)		
Not better and worse	601 (0.15)	184 (0.10)	417 (1.20)		
Sequela	114 (0.03)	47 (0.01)	67 (0.20)		
Death	39 (0.01)	0 (0.00)	39 (0.10)		

### Factors affecting the SADRs in different levels of medical institutions

Table 3 shows the factors that affect the SADRs in primary and nonprimary medical institutions. The elderly, who suffer from multiple diseases, have multiple drug behaviors, have a clear history of ADRs, and use injections and patients with ADRs with duration of more than three days and new ADRs patients are likely to have SADRs in all medical institutions. In addition, patients who use proprietary Chinese medicines in primary and nonprimary medical institutions have high and low SADR risks, respectively. Similarly, patients with a history of illness and surgery have less risk of seeing a doctor in primary medical institutions than that in nonprimary medical institutions.

**Table 3:** SADRs in primary and non-primary medical institutions.

Primary health institutions	Yes OR (95% CI)	No OR (95% CI)
**Gender (refer to male)**		
Female	0.956 (0.91–1.003)	1.034* (1.004–1.064)
**Age (refer to 18–35)**		
0–6	0.848* (0.742–0.969)	0.950 (0.884–1.022)
6–18	0.912 (0.805–1.034)	1.208** (1.109–1.317)
35–60	1.082* (1.005–1.166)	1.324** (1.262–1.389)
60–80	1.145** (1.062–1.234)	1.496** (1.427–1.570)
> 80	1.225** (1.078–1.392)	1.341** (1.253–1.435)
**Number of diseases (refer to 1)**		
≥ 2	1.394** (1.274–1.525)	1.207** (1.166–1.250)
**Polypharmacy (refer to no)**		
yes	2.708** (1.613–4.548)	3.332** (2.963–3.747)
**Type of drug (refer to chemical compound)**		
Chinese patent medicine	1.079** (1.022–1.139)	0.936** (0.899–0.975)
Imported and biological product	1.173 (0.990–1.390)	1.392** (1.311–1.478)
**Past history (refer to no)**		
Yes	1.625** (1.222–2.161)	1.884** (1.754–2.025)
Unknown	0.789** (0.753–0.827)	1.043** (1.011–1.075)
**Past behavior (refer to no)**		
Smoking and drinking	1.333** (1.242–1.430)	1.198** (1.136–1.263)
Allergy	0.875 (0.643–1.190)	1.080 (0.982–1.189)
History of illness and surgery	0.964 (0.752–1.234)	1.304** (1.197–1.420)
**Medication (refer to oral)**		
Injection	7.353** (6.947–7.783)	3.335** (3.205–3.469)
Others	1.016 (0.742–1.392)	1.054 (0.936–1.188)
**Time from medication to ADR (refer to < 3)**		
≥ 3	1.368** (1.221–1.533)	2.312** (2.241–2.385)
**Season (refer to spring)**		
Summer	1.185** (1.109–1.265)	1.033 (0.993–1.074)
Autumn	1.387** (1.304–1.476)	1.193** (1.150–1.238)
Winter	1.560** (1.439–1.691)	1.135** (1.085–1.186)
**ADRs occurrence (refer to first)**		
Again	0.768 (0.569–1.037)	0.870** (0.792–0.956)
**New adverse reactions (refer to no)**		
Yes	1.597** (1.524–1.673)	1.385** (1.342–1.429)

### ADRs in different levels of medical institutions lead to results

Table 4 reports the prognostic results of ADRs among all patients in different levels. Suffered from multiple diseases, have multiple medications, smoking or drinking, injection, occured in autumn and winter, with a clear history of ADRs and long time interval between medication and ADRs cases have poor prognosis in all medical institutions. The risk of poor prognosis for patients using imported drugs or biologic in primary medical institutions is lower than that of patients using general compound drugs, but the risk of poor prognosis for new ADRs is higher than that of the normal groups, and this conclusion in nonprimary medical institutions is opposite. In addition, the prognosis of ADRs in nonprimary medical institutions in other ways of medication is poor.

**Table 4:** ADRs in primary and non-primary medical institutions lead to results.

Primary health institutions	Yes OR (95% CI)	No OR (95% CI)
**Gender (refer to male)**		
Female	0.984 (0.880–1.021)	1.078** (1.034–1.124)
**Age (refer to 18–35)**		
0–6	0.804* (0.647–0.999)	0.669** (0.598–0.749)
6–18	0.854 (0.701–1.041)	0.887 (0.780–1.009)
35–60	0.972 (0.870–1.087)	1.015 (0.952–0.082)
60–80	1.075 (0.960–1.204)	1.122** (1.052–1.196)
> 80	1.373* (1.140–1.654)	1.029 (0.935–1.133)
**Number of diseases (refer to 1)**		
≥ 2	1.651** (1.449–1.881)	1.197** (1.139–1.259)
**Polypharmacy (refer to no)**		
Yes	5.145** (2.862–9.248)	4.004** (3.502–4.579)
**Type of drug (refer to chemical compound)**		
Chinese patent medicine	0.805** (0.736–0.880)	0.639** (0.596–0.685)
Imported and biological product	0.654* (0.453–0.943)	1.200** (1.107–1.302)
**Past history (refer to no)**		
Yes	2.671** (1.870–3.813)	2.237** (2.036–2.459)
Unknown	1.068 (0.995–1.146)	1.246** (1.194–1.301)
**Past behavior (refer to no)**		
Smoking and drinking	1.215** (1.090–1.354)	1.511** (1.408–1.623)
Allergy	1.620** (1.130–2.322)	1.092 (0.951–1.254)
History of illness and surgery	0.916 (0.614–1.368)	1.574** (1.408–1.760)
**Medication (refer to oral)**		
Injection	1.938** (1.804–2.082)	1.188** (1.136–1.243)
Others	2.096** (1.627–2.701)	0.917 (0.799–1.054)
**Time from medication to ADR (refer to < 3)**		
≥ 3	2.345** (2.073–2.653)	3.686** (3.539–3.839)
**Season (refer to spring)**		
Summer	1.008 (0.912–1.114)	1.033 (0.976–1.093)
Autumn	1.145** (1.043–1.257)	1.099* (1.041–1.161)
Winter	1.350** (1.196–1.523)	1.186** (1.114–1.264)
**ADRs occurrence (refer to first)**		
Again	0.505* (0.291–0.879)	1.011 (0.895–1.142)
**New adverse reactions (refer to no)**		
Yes	1.325** (1.230–1.426)	0.872** (0.828–0.918)

*OR* odds ratio, *CI* confidence interval.

*p < 0.05, **p < 0.01.

## Discussion

This study is a retrospective analysis of a regional section within the database of the spontaneous reporting system of Henan Province. The number of ADRs reported in Henan Province has nearly doubled from 2016 to 2020, and the proportion of SADRs has gradually increased, reaching about 10%. In the future, increased adverse events may occur, and drug safety is facing remarkable challenges^14,15^.

The results of the present research suggest that general factors, such as age, disease, type of drug, medication way, new and recurring ADRs and multiple medications, increase the probability of SADRs. Once SADR occurs, it is likely to lead to aggravation of the patient’s original illness and sequelae, which can lead to death in severe cases^16–19^. All 39 deaths are caused by SADRs. The causes of death are hemorrhages and organ failure^20^. The present study has also found that high-level medical institutions have a high proportion of SADRs although people generally think that these institutions are standardized and safe^21^, and this may be due to the fact that high-level medical institutions use multiple drugs and newly developed drugs more commonly. Season is also a factor that should be paid attention. The proportion of SADRs increased significantly in autumn and winter. It may be that the autumn and winter seasons are more susceptible to influenza, and the patient's physique is weaker than other seasons during this period^22,23^.

Overall, this research on primary and nonprimary medical institutions in Table 3 shows that the risk of SADRs in people with multiple medications and having experienced ADRs is quite high. This result further proves the danger of multiple medications and reminds medical staff that they should take special care of patients who have ADRs^24^. However, different factors cause different results in different levels of medical institutions. For example, patients who use proprietary Chinese medicines in primary medical institutions have a higher risk of SADRs than those who use compound drugs, but the opposite is true in nonprimary medical institutions. This finding may be because the indications of traditional Chinese medicine preparations are wide, and the ADRs of some raw materials are not yet clear. The primary medical institutions cannot fully grasp this information^25^. Injection, as one of the risk factors for SADRs, is evident at the primary level^26,27^. This finding reminds primary medical institutions of the need to strengthen the management of proprietary Chinese medicines and injections^28^. Primary medical institutions pay more attention to patients who are not sure whether they have SADRs and history of drug allergies than other hospitals. Their risk of SADRs is lower than that in normal people, which may be the result of the combination of drug use methods in primary medical institutions and attention to special populations^29^.

The report on the prognosis of ADRs is similar to those of previous studies. All medical institutions’ patients with multiple medications, history of ADRs, injection, smoking and drinking and patients with a time interval more than three days have worse prognosis^24,30,31^. This report also found the probability of poor prognosis in nonprimary medical institutions increases in winter. This phenomenon may be due to the shortage of medical beds and insurance funds at the end of the year^21^. In addition, this study has found that although the risk of SADRs caused by minor is high, the prognosis of ADRs in minors is better than adults. This finding shows that medical institutions should pay attention to the risks of the elderly patient. In particular, non-primary medical institutions need to pay more attention to the elderly (> 80 years old) patients because these institutions undertaked more tasks^32^.

The prognosis of patients in primary medical institutions who use other methods of drug delivery is worse because of the lack of professionals or equipment. Patients with drug allergies have worse prognosis^33^. This finding may be the lack of the corresponding training in primary medical institutions. Compared with non-primary institutions, the prognosis of patients with recurrent ADRs is better, indicating that the primary medical institutions are more cautious towards patients with a history of ADRs, however, the prognosis of patients with new ADRs is worse in primary medical institutions and better in non-primary institutions, and it may be due to the fact that primary medical institutions lack effective response measures to new ADRs.

## Limitations

This study has several limitations. First, due to the limitation of data source, the study uses the database of Henan Province, which does not necessarily represent the true situation of the whole country. Second, the database is large and difficult to clean. This study has not analyzed the specific drugs and symptoms that cause ADRs. Third, some recorded ADRs information is not complete, and there are a few missing values in some indicators, which may cause a certain degree of bias. Fourth, due to the insufficient content of the original database, this study has not specifically analyzed the relationship between ADRs and drugs used. Finally, And media attention and recent publication of an ADR in the literature might affect the reporting behaviors^34^.

## Conclusion

This study analyzes the influencing factors and countermeasures of ADRs. The absolute number of SADRs is increasing, and a high proportion occurs in nonprimary medical institutions. Patients with multiple medications, history of ADRs, and the interval between medication and ADRs exceeding three days have high risk of SADRs and poor prognosis. Other factors lead to different results in different levels of medical institutions. We suggest strengthening the supervision of proprietary Chinese medicines and injections; Introduce a plan to deal with new ADRs in primary medical institutions and paying attention to the safety and health status of patients with history and used imported drugs and biologic with caution in nonprimary medical institutions.
