# Author Correction: The severity of adverse drug reactions and their influencing factors based on the ADR monitoring center of Henan Province

**DOI:** 10.1038/s41598-022-06561-5

**Published:** 2022-02-08

**Authors:** Ziqi Yan, Zhanchun Feng, Zhiming Jiao, Chaoyi Chen, Ganyi Wang, Da Feng

**Affiliations:** 1 School of Medicine and Health Management, Tongji Medical College, Huazhong University of Science and Technology, Wuhan, 430030 Hubei China; 2 Medical Products Administration and Center for ADR Monitoring of Henan, Zhengzhou, 450008 Henan China; 3 School of Pharmacy, Tongji Medical College, Huazhong University of Science and Technology, Wuhan, 430030 Hubei China

Correction to: *Scientific Reports* 10.1038/s41598-021-99908-3, published online 14 October 2021

The original version of this Article contained an error in the Acknowledgments section.

“The authors would like to thank the Henan Adverse Drug Reaction Monitoring Center for providing the data. The data in this study is publicly available and accessible. The information in this paper does not represent the opinion of Henan Adverse Drug Reaction Monitoring Center or national centres. The National Natural Science Foundation of China (No. 72074088) and the Young Scientists Fund (No. 72804052) supported this research. We would like to thank the National Natural Science Foundation of China and the Young Scientists Fund for the funding for this research.”

now reads:

“The authors would like to thank the Henan Adverse Drug Reaction Monitoring Center for providing the data. The data in this study is publicly available and accessible. The information in this paper does not represent the opinion of Henan Adverse Drug Reaction Monitoring Center or national centres. The National Natural Science Foundation of China (No. 72074088) and the Young Scientists Fund (No. 71804052) supported this research. We would like to thank the National Natural Science Foundation of China and the Young Scientists Fund for the funding for this research.”

The original Article has been corrected.

